# Transperineal Ultrasound-Guided 12-Core Prostate Biopsy: An Extended Approach to Diagnose Transition Zone Prostate Tumors

**DOI:** 10.1371/journal.pone.0089171

**Published:** 2014-02-25

**Authors:** Ming-Hua Yao, Li-Ling Zou, Rong Wu, Le-Hang Guo, Guang Xu, Juan Xie, Pei Li, Shuai Wang

**Affiliations:** 1 Department of Ultrasound in Medicine, Shanghai Tenth People's Hospital, Tongji University School of Medicine, Shanghai, China; 2 Department of Health Statistics, Tongji University School of Medicine, Shanghai, China; National Cancer Center, Japan

## Abstract

**Objective:**

Transperineal ultrasound-guided (TPUS) 12-core prostate biopsy was evaluated as an initial strategy for the diagnosis of prostate cancer, The distribution of prostate cancer lesions was assessed with zone-specific biopsy.

**Methods:**

From January 2010 to December 2012, 287 patients underwent TPUS-guided 12-core prostate biopsy. Multiple cores were obtained from both the peripheral zone (PZ) and the transition zone (TZ) of the prostate. Participants' clinical data and the diagnostic yield of the cores were recorded and prospectively analyzed as a cross-sectional study.

**Results:**

The diagnostic yield of the 12-core prostate biopsy was significantly higher compared to the 6-core scheme (42.16 vs. 21.6%). The diagnostic yield of the 10-core prostate biopsy was significantly higher compared to the 6-core scheme (37.6 vs. 21.6%). The 12-core scheme improved the diagnostic yield in prostates >50 ml (12-core scheme: 28.1% vs. 10-core scheme: 20.4%; p = 0.034).

**Conclusions:**

The 12-core biopsy scheme is a safe and effective approach for the diagnosis of prostate cancer. TZ biopsies in patients with larger prostates should be included in the initial biopsy strategy.

## Introduction

Prostate cancer is the sixth leading cause of cancer-related death among older men in developed countries [1] and is on the rise in developing countries including China. Its pathogenesis is poorly understood. Prostate cancer are often asymptomatic during the early stages of disease. Although PSA-based screening has resulted in a significant increase in the detection rate of PCa, its use remains controversial because elevated levels of PSA are not cancer specific. Moreover, clinically significant PCa can exist in men with relatively low PSA levels [2](Thompson et al, 2004). Besides the conventional digital rectal examination (DRE) and the controversy surrounding prostate specific antigen (PSA)-based screening, ultrasonography (US) and magnetic resonance imaging (MRI) are the most common imaging technologies to screen for prostate cancer. US can visualize the prostate directly; and due to certain advantages— it is conducted in real-time, it is portable and economical— it is often used for biopsy guidance. MRI can provide more information about the properties of the tissue, such as enhancement and diffusion, that are valuable in the evaluation of tumor extent [3]. According to the guidelines of the European Association of Urology (EAU), among the main diagnostic tools to diagnose prostate cancer, the systematic prostate biopsy under ultrasound guidance is the preferred diagnostic method [4]. An ultrasound-guided biopsy uses either a transrectal or transperineal approach to access the prostate. Although both have been reported to have equal detection rates [5]–[7], the transperineal approach may be preferred under certain circumstances [8]. Even though few biomarkers exist, biopsy is the most successful diagnostic approach [9]. TPUS-guided biopsy provides uniform sampling of the entire prostate and a relatively high probability of clinical diagnosis [10]. However, the search for an improved biopsy technique, which includes a better diagnosis with relatively few complications, is ongoing [11]. Biopsy techniques that optimize the number of cores that are sampled, as well as their locations within the prostate gland, may be considered [12]. In this prospective analysis, we estimated the diagnostic yield of different biopsy schemes, analyzed the locations within the prostate of the carcinoma-positive cores identified during TPUS-guided extended biopsy, and evaluated the efficacy of TPUS-guided extended biopsy for detecting disease in various locations within the prostate gland.

## Materials and Methods

### Subjects

From January 2010 to December 2012, 287 patients underwent TPUS-guided biopsy at our hospital, Department of Ultrasound in Medicine, Shanghai tenth People's Hospital. Inclusion criteria were one or more of the following: i) High PSA level (>4 ng/ml), but urinary tract infection, prostatitis or prostate massage excluded; ii) Abnormal findings by DRE; iii) Hypoechoic areas during examination of the prostate by transrectal or abdominal ultrasound; iv) Abnormalities identified by magnetic resonance imaging (MRI) of the prostate (such as T2-weighted). Patients with previous histology requiring repeat biopsy were excluded from the study. This study was performed in strict accordance with the ethical guidelines of the Helsinki Declaration. The study protocol was approved by the Ethics Committees of the People's Hospital of Tongji University, Shanghai, and all participants provided written informed consent. Patients were divided into three groups according to age, prostate volume, and PSA level. Patient demographic and clinical data were recorded as well as the numbers of carcinoma-positive cores by location within the prostate. The diagnostic yield of the 12-core method was compared to the sextant biopsy and 10-core biopsy schemes.

### Equipment

The Hitachi 8500 sonographer (Hitachi, Japan) equipped with a 5.0/7.5 MH z transrectal dual-plane probe was used to perform ultrasonography. The Bard automated biopsy gun (Tempe, Arizona, America) with 22 mm range and 18G needle was used for biopsy.

### Biopsy

**Figure 1:**
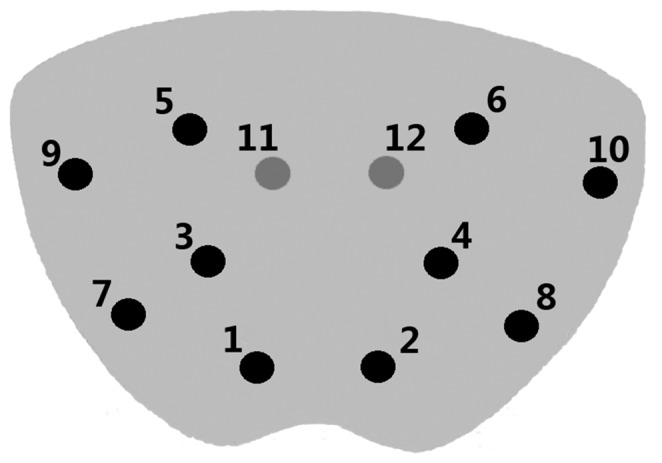
Transverse section: Biopsy cores were distributed in pairs. 1 to 6: the standard sextant cores; 7 to 10: the four additional cores in the lateral peripheral zone (PZ); 11 to 12: two cores in the transition zone (TZ).

Preoperative examinations including routine blood and urine tests were performed to exclude coagulation disorders, hematuria, and urinary tract infections. The 12-core transperineal prostate biopsy was carried out with patients in the lithotomy position. Perineal skin was prepared, disinfected, and locally anesthetized with 1% lidocaine. Under the guidance of TRUS, the 18G biopsy needle was inserted through the perineal skin, and the cores were taken as follows: traditional sextant; four cores in the lateral PZ; two cores in the TZ (Figure 1). After biopsy, specimens were collected in 10% formaldehyde for pathological examination. Post-surgical pain was assessed using the visual analogue scale (VAS: a score of 0 indicated no pain, a score of 10 indicated extreme pain). Post biopsy, patients remained in the hospital for observation for two days. Patients were followed-up to gather information relating to biopsy-related complications by telephone for one week.

### Statistical analysis

SPSS 17 was used for statistical analysis. χ^2^ test was used to compare the diagnosis rate among groups; p<0.05 was considered statistically significant.

## Results

287 patients were enrolled in the study. Table 1 shows the baseline characteristics for all patients. 42.1% (121/287) patients tested positive for prostate cancer by 12-core biopsy; 37.6% (108/287) patients tested positive for prostate cancer by 10-core biopsy; and 21.6% (62/287) patients tested positive for prostate cancer by 6-core biopsy. Diagnostic yield among the three approaches was significantly different (p<0.01; Table 2). After the pathological examination, the Gleason score (GS) of each patient was obtained. The results show that 65 patients (53.8%) had a GS of 6; 44 patients (36.3%) had a GS of 7; and 12 patients (9.9%) had a GS of 8 to 10.

**Table 1:** Patient baseline characteristics (n = 287).

Criteria	Value
Median Age, in years (range)	71 (25–86)
Age>60	86.4%
Mean Prostate volume, ml, (SD)	47.0 (23.0)
Prostate volume>50 ml	36.2%
Mean Level of PSA, ng/ml, (SD)	22.8(29.5)
Level of PSA>10 ng/ml	48.1%

**Table 2:** Diagnostic yield of TPUS-guided sextant, 10-core, and 12-core biopsy schemes.

	Positive Diagnosis		Negative Diagnosis		Total
Technique	Number	Percentage	Number	Percentage	Number
Sextant biopsy	62	21.6	225	78.4	287
10-core biopsy	108	37.6*	179	68.4	287
12-core biopsy	121	42.1*	166	57.9	287

*p<0.01: statistically significant difference between the schemes

The cancer lesions of 121 patients were characterized by real-time ultrasonography. The maximal diameter of the lesion was less than 10 mm in 15 patients (12.4%); between 10 and 15 mm in 22 patients (18.2%); and greater than 15 mm in 24 patients (19.8%). Diffusion lesions were observed in 29 patients (24.0%), and the lesion was unobservable in 31 patients (25.6%).

There was no statistical difference in the diagnostic yield of carcinoma-positive cores sampled from the PZ by sextant biopsy compared to 10-core biopsy (p = 0.54, Table 3), which indicates that there is a uniform distribution of lesions in the PZ. There were no significant differences in diagnostic yield by 10-core and 12-core biopsy schemes when grouped by age, PSA levels, and DRE. When patients were grouped according to prostate volume (≤50 ml and >50 ml), 12-core biopsy diagnosed significantly more patients with carcinoma-positive cores in the TZ compared to 10-core biopsy (28.1 vs. 20.4%, Table 4).

**Table 3:** Diagnosis of carcinoma positive cores by location within the prostate gland: sextant and 10-core biopsy schemes.

Core Number	Lobe	Site	Positive number	%	p*
1	Right	Apex	41	33.88	0.54
2	Left	Apex	42	34.71	
3	Right	Midgland	43	35.54	
4	Left	Midgland	38	31.40	
5	Right	Base	45	37.19	
6	Left	Base	42	34.71	
7	Right	Lateral midgland	37	30.58	
8	Left	Lateral midgland	32	26.45	
9	Right	Lateral base	30	24.79	
10	Left	Lateral base	40	33.06	

*p = 0.54: no significant difference in diagnosis by sextant or 10-core biopsy

**Table 4:** Group analysis of prostate cancer diagnosis using 10-core and 12-core TPUS guided biopsy schemes.

	10-core biopsy		12-core biopsy		*p value*
	number	percentage	number	percentage	
Age (in years)					0.942
≤60	6	5.6	8	6.6	
61∼80	78	72.2	87	71.9	
≥81	24	22.2	26	21.5	
Prostate volume					0.034*
≤50 ml	99	79.6	99	71.9	
>50 ml	9	20.4	22	28.1	
Level of PSA					0.732
4∼10 ng/ml	25	23.1	28	23.1	
10∼50 ng/ml	37	34.3	47	38.8	
≥50 ng/ml	46	42.6	46	38.0	
DRE					0.857
Positive	17	15.7	18	14.0	
Negative	91	84.3	103	86.0	

* p value  = 0.034 Rate of prostate cancer diagnosis is improved by TPUS-guided 12-core biopsy in patients with larger prostates

Participants were similarly tolerant of procedural and post-procedural pain (VAS score of 0-3). Gross hematuria was observed in 21 patients (7.3%), hematuria with hematospermia in 8 patients (2.8%), urinary tract infection in 19 patients (6.6%), and acute urinary retention in 3 patients (1.0%). No serious biopsy hematoma or neurovascular injury occurred.

## Discussion

Prostate cancer is the most common malignant tumor among older men [13]. According to a study [14], the median age of diagnosis of prostate cancer in the United States is more than 65 years. Prostate cancer reduces life expectancy and lowers quality-of-life[15], [16]. TPUS-guided biopsy is the preferred approach for prostate cancer diagnosis. The traditional sextant TPUS biopsy has a history of under-diagnosing a large number of patients [17]. It is likely that more cores should be sampled to increase the diagnostic yield. However, such approaches may lead to a greater number of biopsy-associated complications. It is essential to achieve a balance between diagnostic yield, the number of cores sampled, and the risk for complications.

Since its introduction by Hodge in 1989, sextant biopsy has been modified several times [18]. In particular, the sextant protocol fails to detect a large number of carcinomas in the PZ region of the prostate gland [19]. This observation prompted the Italian National Comprehensive Cancer Network to recommend the sampling of 4 cores from the lateral PZ in addition to the traditional sextant scheme [20]. In current practice, 10–12 cores are used as an extended biopsy approach [21], with minimal biopsy-related complications [22]. Previous reports of the results of the 12-core biopsy were based on the conventional sextant plus 6 cores in the peripheral zone. The detection rate ranged from 30.3% to 51% [23], [24]. Some researchers believe that there is no difference in the detection rate between a 6-core and 12-core biopsy [25], or between an 8-core and 12-core biopsy [26], while other researchers believe that adding more cores will increase the detection rate [27], [28], [29]. Moreover, some studies focused on the relationship between the Gleason score and the 12-core biopsy. For instance, Arrabal-Polo et al. determined that there is no difference in Gleason score between the specimens from 6-core and 12-core biopsies [30]–[34]. Our study indicated that the TPUS-guided 12-core biopsy technique provided improved diagnostic yields, especially in cancers originating in the TZ of larger prostates, without increasing the risk of biopsy-related complications.

Anatomically the prostate is divided into three regions: the PZ, TZ and central zone (CZ), and is covered in a tough fibrous sheath [35]. The PZ is further divided into the apex, midgland and the base. Results describing the distribution of tumors within the zones of the prostate are inconsistent. Some studies suggest that 75% prostate cancers occur in the PZ, and 25% occur in the CZ and TZ [36]. A Study by Demura et al, [37] indicated that the distribution of prostate cancer is uniform within the entire gland. Other reports show that the carcinoma foci are more localized in the anterior location [38]. Our study revealed that there are no differences in tumor distribution between the apex, midgland and base in the PZ. However, although not statistically significant, the ratio of the diagnostic yield of 10-core biopsy relative to sextant biopsy was 1.74 (37.63% vs 21.60%), indicating that increasing the number of cores sampled in the PZ may improve diagnosis.

Age is a strong predictor of prostate cancer. Several reports [39], [40] suggest that mortality is positively correlated with age. In our study, the age of the majority of patients diagnosed with prostate cancer was over 60 years (>90%). In these older males, the diagnostic yield was similar with both the 10-core and 12-core biopsy schemes, suggesting that the number of cores is not an important predictor of the success of the diagnostic approach in this patient population.

Prostate volume is another predictor of prostate cancer. Yamamoto et al.[41] and Novara et al. [42] showed that there was a negative correlation between diagnostic yield and prostate volume. In our study, the diagnostic yield of 12-core biopsy was higher than that of 10-core biopsy, and when prostate volume was over 50 ml, adding cores in the TZ significantly increased the number of tumors detected.

PSA is a protease secreted by prostate epithelial cells. A number of events cause an increase in PSA levels, including benign prostate hyperplasia (BPH) and prostatitis [43]. Currently, it is recommended not to screen for prostate cancer based on PSA-levels due to the risks of over-diagnosis and over-treatment [44], [45]. However, the predictive value of PSA is an important non-invasive screening method. Screening for prostate cancer based on PSA significantly reduces the risk of metastatic cancer [46]. Our data indicate that biopsy should be performed in patients with PSA levels greater than 4 ng/ml, but extended approaches are not required.

Several reports [47], [48] show that the diagnostic yield from core samples in the TZ is so low that it could be omitted. Other studies recommend including biopsy cores from the TZ to improve the diagnostic yield [49], at least in repeat biopsies [50]. Our research found that the addition of 2 cores in the TZ resulted in the diagnosis of 13 extra TZ-only positive patients who were DRE negative. Further research based on a larger number of patients is required before it can be concluded that the TZ should be routinely included in prostate biopsy schemes.

## Conclusion

In summary, prostate lesions occur uniformly in the PZ. Biopsy of the TZ should be reserved as initial biopsy in patients whose prostate volume is over 50 ml. The 12-core biopsy approach maximizes the sensitivity of cancer detection while keeping biopsy-related complications low. Thus, TPUS-guided 12-core biopsy is a safe and effective method to improve the diagnostic yield of tumors occurring in the TZ of larger prostates.
